# The Confluence of HIV-1 and HIV-2: Implications for Disease Progression and Insights for Therapy

**DOI:** 10.1155/ijm/3145677

**Published:** 2025-07-10

**Authors:** Edwin Magomere, Charles Ochieng' Olwal, Becky Ewurama Tetteh, Mark Appeaning, Thumbi Ndung'u, George Boateng Kyei, Peter Kojo Quashie

**Affiliations:** ^1^West African Centre for Cell Biology of Infectious Pathogens, College of Basic and Applied Sciences, University of Ghana, Accra, Ghana; ^2^Department of Biochemistry, Cell and Molecular Biology, University of Ghana, Accra, Ghana; ^3^Yemaachi Biotech, Accra, Ghana; ^4^Africa Health Research Institute, University of KwaZulu Natal, Durban, South Africa; ^5^HIV Pathogenesis Programme, The Doris Duke Medical Research Institute, University of KwaZulu-Natal, Durban, South Africa; ^6^Ragon Institute of MGH, MIT and Harvard University, Cambridge, Massachusetts, USA; ^7^Division of Infection and Immunity, University College London, London, UK; ^8^Department of Virology, Noguchi Memorial Institute for Medical Research, University of Ghana, Accra, Ghana; ^9^University of Ghana Medical Center (UGMC), University of Ghana, Accra, Ghana

**Keywords:** attenuated progression, cocirculation, disease progression, dual infection, HIV-1 and HIV-2, therapeutics, viral interactions

## Abstract

Two distinct types of human immunodeficiency virus (HIV), namely, HIV-1 and HIV-2 exist. HIV-1 is responsible for the global pandemic and has an aggressive pathogenesis. On the contrary, HIV-2 is not only less aggressive but also confined to West and Central African regions. Even after four decades of HIV research, a scalable cure or effective vaccine against HIV remains elusive. Consequently, the concept of a functional cure or vaccine, targeting to limit disease progression, allowing sufficient time for the immune response to clear the virus, has gained traction. Efforts to identify new therapeutic targets for development of a functional cure have focused on elite controllers, that is, individuals who naturally control HIV-1 infection in the absence of antiretroviral therapy. However, little progress has been associated with these efforts perhaps due to the scarcity of elite controllers, who make up only 0.15% of HIV-1 infected population globally. A distinct but largely unexplored subset of HIV patients comprise HIV-1 and HIV-2 dually infected individuals. This group of patients naturally presents with an attenuated disease progression phenotype akin to natural controllers. In this review, we discuss the attenuated disease progression phenomenon in dually infected individuals and offer potential explanations for this unanticipated observation. Additionally, we propose potential therapeutic and/or vaccine strategies that could leverage interactions of HIV-1 and HIV-2. Such strategies are likely to inform alternative therapeutics. A thorough understanding of the mechanism underlying the attenuated disease progression phenotype in HIV dually infected individuals is crucial for the design of a functional cure.

## 1. Background

Although there has been a decline in the transmission of human immunodeficiency virus (HIV) post-antiretroviral therapy (ART) era, the virus remains a major global health challenge [1–3]. HIV-related diseases have claimed about 40.1 million lives since it is discovery in the early 1980s [4]. Approximately 39.9 million people across different regions were living with HIV as at the end of 2023 [5]. Sub-Saharan-Africa (sSA) bears the greatest brunt of the disease with approximately 25.7 million cases [3]. Eastern and Southern Africa regions are the most severely affected, accounting for more than 20 million (78%) of the 25.7 million cases in sSA [6].

There are two distinct types of HIV, namely, HIV-1 and HIV-2. Although HIV-1 is responsible for the largest proportion of global acquired immune deficiency syndrome (AIDS) pandemic, HIV-2 is an important contributor to the pandemic in some regions [7, 8]. About two million people are living with HIV-2, mainly in the West and Central Africa regions [8]. While the available data does not explicitly describe the current prevalence of HIV-2, a number of cases have been reported in countries with close ties to West Africa, including European, Asian, and North American countries [9, 10]. Portugal, in particular, reported a high prevalence rate of 5.4% [11].

Dual infection with both HIV-1 and HIV-2, hereafter referred to as HIV-dual, make up approximately 1% of HIV infections in West and Central African regions [12–15]. Despite both types of HIV being endemic in West and Central Africa, the overall HIV prevalence is lower in these regions compared to other parts of Africa. This phenomenon may be partly due to cocirculation of both HIV types. There is, thus, a potential interplay between HIV-1 and HIV-2 that limit establishment, spread, and/or progression of HIV during dual infection. In this review, we delve into the dynamics of coinfection of HIV-1 and HIV-2 and discuss how direct and indirect interactions between these two viruses could influence disease trajectory and clinical outcomes, as well as potential implications for therapeutics and vaccine design.

## 2. Epidemiology of HIV-1 and HIV-2 Infections

The overlap in circulation of both HIV-1 and HIV-2 in West and Central African regions sometimes results in sequential or simultaneous infection with both viruses. In contrast, HIV-dual infections are rare in other regions of Africa. The majority of HIV-2 cases reported outside Africa have epidemiological links to the West African epidemic, implying that these cases originated from West Africa and spread out to other continents [16]. To date, there are no confirmed reports of HIV-2 transmissions in other parts of Africa. This exclusive geographical distribution of HIV-2 in Africa could be driven by either host or viral factors yet to be uncovered. Notably, different HIV-1 subtypes also show exclusive patterns of distribution, with subtypes C and D dominating the epidemics in Southern and Eastern Africa while accounting for less than 0.1% of the Central and West African epidemic [17], which is predominated by a circulating recombinant form (CRF02_AG). Additionally, HIV-1 subtypes vary in transmission rate and disease severity, as reflected by the high prevalence rate of Subtypes C and D (6.8%) in Southern and Eastern Africa subregions [18, 19]. On the contrary, the West and Central African HIV epidemic is milder, characterized by a relatively lower prevalence (approximately 1.9%) [19].

Of the 8 HIV-2 subtypes identified by surveillance of viral molecular evolution, only two (i.e., Subtypes A and B) established an ongoing epidemic in the West African population [20]. Guinea Bissau was designated as the epicenter of the HIV-2 epidemic, with the highest prevalence being reported in women older than 45 years [13]. The majority of these women maintained low viral load and high CD4^+^ T-cell counts [13]. Compared to HIV-1, HIV-2 is associated with fewer deaths (reviewed by [21]). The most recent reported prevalence of HIV-2 in Guinea Bissau is about 2.8% [22]. However, the estimation of the HIV-2 prevalence rate could be confounded by the limited attention towards HIV-type specific diagnoses. Most new diagnoses are reported as HIV positive, without further typing tests since treatment modalities are practically the same for both viral types.

The prevalence of HIV-1 in Guinea Bissau increased from 0.5% to 3.6% over the period when HIV-2 prevalence declined [23], suggesting an inverse relationship. Although the drivers of such an unlikely relationship are not entirely understood, it is thought that mechanisms, such as a decrease in viral fitness, lower transmission efficiency, and competitive exclusion of HIV-2 through negative dominance, could explain this phenomenon [24]. Evidence from phylogenetics modeling predicted that up to 30% of the decline in HIV-2 prevalence when HIV-1 is in circulation is due to competitive exclusion while the remaining 70% was attributed to population-level sociobehavioral interactions [25, 26].

Sporadic cases and small clusters of HIV-2 have been reported in countries that had close trade, historical and colonial links to West Africa. In Europe, Portugal is leading with the highest HIV-2 prevalence of 5.4% [27]. The epidemic in Portugal was linked to the historical and migratory ties with Guinea Bissau, which coincidentally has reported the highest number of cases in West Africa [11, 28, 29]. According to WHO, HIV-2 accounts for approximately 1% of all HIV cases in France [30]. There is strong phylogeographic evidence linking HIV-2 cases in France to former colonies in West Africa including Côte d'Ivoire and Senegal [16]. HIV-2 epidemic in Spain is declining with the current number of cases being below 10 cases per year [31]. Generally, HIV-2 prevalence outside West Africa is declining and is mainly diagnosed among migrants of Sub-Saharan Africa [31].

## 3. Differences and Similarities in Pathophysiology of HIV-1, HIV-2, and HIV-Dual Infection

Although HIV-1 and HIV-2 share many similarities, such as modes of transmission and replication [32], their pathophysiology exhibits key differences (Table 1). In addition, the pathophysiology of HIV-dual infection differs from that of monoinfections as discussed below.

HIV-2 infection is characterized by a longer asymptomatic phase [46], slower CD4 decline [45], and lower plasma viral loads compared to HIV-1 [13, 47]. Disease progression in HIV-1 infected individuals is slower in HIV-dual infections relative to HIV-1 monoinfection [48]. All together, these features contribute to a lower overall mortality rate among HIV-2 and HIV-dual [13, 47]. Without treatment, only 30% of HIV-2 patients progress to AIDS and death [13]. However, once CD4 count drops to below 200 cells/microliter, the risk of death in HIV-2 and HIV-dual is similar to that of HIV-1 monoinfection [7].

HIV-2 patients exhibit higher CD4 percentages during acute infection (i.e., 28% in HIV-2 vs. 22.3% in HIV-1) and experience a slower annual decline in CD4 percentages (0.4% in HIV-2 vs. 0.9% in HIV-1) [13, 33]. AIDS symptoms develop at higher CD4 percentages in HIV-2 infections (18.2%) compared to HIV-1 patients (8.2%). Viral loads in HIV-2 are also significantly lower—about 28-fold lower than in HIV-1 infections [13, 33]. These factors collectively result in a longer asymptomatic phase for HIV-2.

Clinical studies have shown that HIV-2-infected individuals survive twice as long as those with HIV-1 (i.e., 15.6 years for HIV-2 vs. 8.2 years for HIV-1), with median time-to-AIDS being much longer (i.e., 14.3 years for HIV-2 vs. 6.2 years for HIV-1) [13, 34]. These observations, largely derived from studies conducted during the pre-ART era, highlight the distinct natural histories of these viruses. The impact of ART on this pattern is yet to be investigated.

Compared to HIV-1, transmission of HIV-2 is less efficient, which explains the significantly fewer cases globally [27]. For instance, mother-to-child transmission rates are much lower in HIV-2 (1%–2%) compared to HIV-1 (15%–30%) [35]. The lower rates of vertical transmission can be linked to the persistently lower viral loads in HIV-2 peripartum women, hence decreased viral shedding during delivery [40].

### 3.1. HIV-Dual Infections and Disease Progression

The influence of HIV-2 on HIV-1 infectivity and disease progression was first reported in a 9-year longitudinal follow-up cohort of Senegalese female sex workers (FSW) [42]. The study involved periodic measurement of seroprevalence and seroincidence, which showed that women infected with HIV-2 gained protection against subsequent HIV-1 infection [42]. While the study revealed a high incidence of other sexually transmitted diseases (STDs) among all study participants, HIV-2 infected women had a lower incidence rate of HIV-1 compared to the HIV-2-seronegative women, with a relative risk of 0.32 (*p* = 0.008) [42]. Understanding the mechanism underlying this potential cross-protection may be directly relevant to HIV-1 vaccine development. Another study showed that coinfection with HIV-1 and HIV-2 increased the probability of AIDS-free survival among dually infected individuals in a long-term follow-up cohort of police officers in Guinea Bissau [48]. Subsequently, it was revealed that HIV-dually infected individuals had an AIDS-free survival of 104 months compared to 68 months in HIV-1 monoinfected and 83 months in HIV-2 mono infected individuals [13].

The long asymptomatic phase in HIV-dual infection is generally characterized by higher CD4^+^ T-cell count and a slow increase in CD8^+^ T-cells [13]. In a study by Esbjörnsson and colleagues, individuals who were infected with HIV-2 prior to HIV-1 had the longest time-to-AIDS and maintained the highest CD4^+^ T-cell counts [48]. This observation suggested that in the presence of HIV-2, the progression of HIV-1 is attenuated. In addition, HIV-1 genetic diversity was lower in participants with HIV-dual infection relative to those with HIV-1 monoinfection at similar time points after infection [48]. Esbjornsson and colleagues showed that HIV-2 potentially influences the disease trajectory of HIV-1, but the mechanisms of this interference remain a jigsaw puzzle.

## 4. Hypotheses Underlying Attenuated Disease Progression in HIV-Dual

### 4.1. Inhibition of T-Cell Activation by HIV-2 Nef

**Figure 1:**
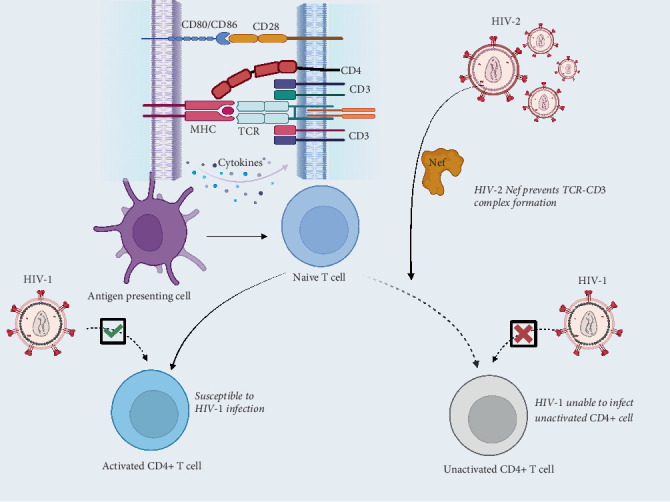
Attenuation of CD4+ T-cell activation by HIV-2 derived negative factor (Nef). HIV-2-derived Nef downregulates CD3-TCR complex formation blocking activation signals. Nonactivated CD4 T-cells are less susceptible to HIV-1 infection. Created in BioRender (https://app.biorender.com/)

In the quest to understand the unique virus-virus interaction that results in slow disease progression in HIV-dual infections, Nyamweya and colleagues suggested the concept of attenuated progression phenotype as an underlying phenomenon [49]. The negative factor (Nef) from simian immunodeficiency virus (SIV), which is closely related to HIV-2, has been implicated in down-modulation of TCR-CD3 complex formation [50], suppressing the responsiveness of T cells to activation (Figure 1).

Inhibition of T-cell activation by HIV-2 Nef has also been demonstrated [51]. This inhibition renders CD4^+^ T-cells less susceptible to subsequent infection by HIV-1 during contemporaneous HIV-1 superinfection [51]. Also, cell-to-cell infection is greatly decreased, slowing down disease progression. Structural analysis of HIV-2 Nef revealed a highly conserved di-leucine sorting motif that binds CD3 endocytosis motif promoting AP-2 mediated CD3 endocytosis [52]. This conserved C-terminal motif is absent from HIV-1, suggesting a potential role in slowing down disease progression among HIV-2 infected individuals.

### 4.2. HIV-2 Induces High *β*-Chemokine Production

**Figure 2:**
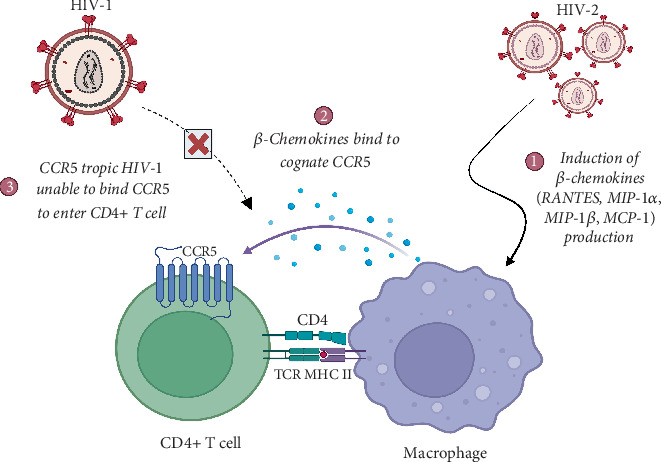
HIV-2 induces over production of *β*-chemokines. These chemokines are the cognate ligands of CCR5 receptors, which competitively occupy their CCR5 receptors preventing R5-tropic HIV-1 viruses from using CCR5 coreceptors to infect cells. Created in BioRender (https://app.biorender.com/).

HIV-2 can alter expression of cellular factors that influence susceptibility of target cells to infection. For instance, an in vitro stimulation of HIV-2 infected lymphocytes resulted in the overexpression of beta-chemokines, the natural ligands of chemokine receptor CCR5, which preferentially bind their cognate receptors, precluding CCR5-tropic HIV-1 from CCR5-mediated cell entry [53] (Figure 2). A recent study demonstrated that infection of monocyte-derived macrophage (MDM) cell line with HIV-1 or HIV-2 resulted in differential induction of CCL2 *β*-chemokine [54]. Elevated levels of these chemokines were observed in MDM infected with HIV-2. Our unpublished data point towards elevated levels of four *β*-chemokines (CCL11, CCL2, CCL3, and CCL5) in plasma of HIV-2 and HIV-dual infections relative to HIV-1 monoinfection (unpublished data). Despite variability in patients, our data support in vitro findings that reported higher plasma levels of *β*-chemokines [54]. Altogether, these reports favor the hypothesis that HIV-2 triggers production of high levels of these chemokines that in turn dampen infectivity of HIV-1 during dual infection.

### 4.3. Cross-Protective Immune Responses

Studies suggest that heterologous T-cell responses between HIV-1 and HIV-2 infections could contribute to slowed HIV-1 disease progression during HIV-dual infection [55]. Gag-specific cross-reactive T-cell responses have been demonstrated [56]. HIV-2 specific CD8^+^ T-cell responses are likely to contribute to attenuated HIV-1 disease progression in HIV-dual infection [46]. In addition, a strong, early-differentiated polyfunctional Gag-specific cytotoxic T-cell response that could potentially target reactivated latent viruses represents a protective cross-reactive immunity [46]. Moreover, neutralizing antibodies raised against HIV-2 can potentially neutralize HIV-1 viruses [57, 58] preventing infection of new CD4^+^ T-cells and potentially slowing down HIV-1 disease progression.

### 4.4. Viral Proteins and Host Restriction Factors

Vpx, an HIV-2 specific protein, has been implicated in reducing HIV-1 infectivity during dual infection [43]. In addition, host viral restriction factors such as sterile alpha motif and histidine-aspartic acid domain–containing protein-1 (SAMHD1) and tripartite motif–containing protein 5 (TRIM5*α*) play a critical role in limiting HIV-1 viral replication [59]. In the subsequent section, we discuss potential mechanisms underlying the antiviral effects of these factors.

#### 4.4.1. HIV-2 and SIV Vpx Degradation of SAMHD1 and Effect on Cytokine Responses

Previous studies have hinted at the possible protective effects of HIV-2 against HIV-1 in HIV-dual infection [42, 60]. Understanding the mechanism of this protective effect remains an elusive task given the variability in cohort subjects and the complicated methodologies involved. To circumvent these barriers, studies have explored the use of VSV-G-pseudotyped HIV-1 and HIV-2 virions to demonstrate interviral interactions at the cellular level. Mahdi and colleagues showed that pretransfection of HEK293T-cells with plasmids coding for HIV-2 Vpx reduced the rate of subsequent transduction with VSV-G pseudotyped HIV-1 vectors [43]. Additionally, Matsuda and colleagues transfected the CD4^+^ human T lymphoid cell line (SUP T1 cell line) with the Vpx^SIVmac^ plasmid and observed significant loss of infectivity of the resultant cell-free virions [61]. Put together, these findings demonstrated that in the presence of Vpx^HIV2^ or Vpx^SIVmac^, HIV-1 infectivity is dampened in both HEK293T and SUPT1 cells [43, 61].

The role of Vpx in neutralizing the antiviral factor SAMHD1 has also been studied extensively [43, 62]. Vpx acts by promoting proteasomal degradation of SAMHD1 through DDB1/DCAF1-dependent E3 ubiquitin ligase [63]. Mohamed and colleagues showed that in addition to enhancing proteasomal degradation of SAMHD1, transfecting Vpx^SIVmac^ and Vpx^HIV2^ into THP-1 and U937 cells resulted in suppression of proinflammatory cytokines, skewing the immune response towards an anti-inflammatory state [64]. While the degradation of SAMHD1 and suppression of the proinflammatory immune response by HIV-2^Vpx^ and SIV^Vpx^ promoted their ability to infect noncycling immune cells such as macrophages [65, 66], the presence of Vpx dampened the infectivity of HIV-1 in vitro [67]. The exact mechanism by which Vpx reduces HIV-1 infectivity is not fully understood, warranting deeper investigation.

**Figure 3:**
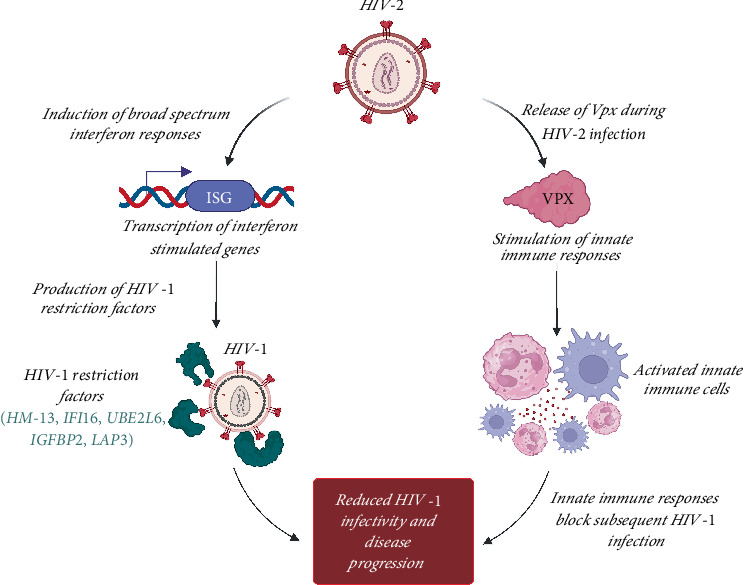
Preinfection with HIV-2 can potentiate innate immune response through Vpx protein. Vpx stimulates innate immune cells potentially blocking subsequent HIV-1 infection. HIV-2 can also induce a broad-spectrum interferon response. These interferons promote production of HIV restriction factors through interferon stimulated gene (ISG). Created in BioRender (https://app.biorender.com/).

Some studies have investigated the ability of Vpx to potentiate innate immune responses (Figure 3) during acute HIV-2 infection potentially limiting subsequent HIV-1 infection [68, 69]. Broad spectrum interferon responses due to preinfection with HIV-2 was linked to protection against a subsequent HIV-1 infection [24]. Interferons could exert their antiviral effects indirectly by stimulating secretion of viral restriction factors through the expression of interferon stimulated genes (ISGs) or directly through interferon-mediated inflammation (Figure 3). Thus, potentiating production of viral restriction factors in a similar fashion as Vpx^HIV2,^ would help to achieve viral remission.

#### 4.4.2. TRIM5*α*-Mediated Capsid (CA) Degradation

**Figure 4:**
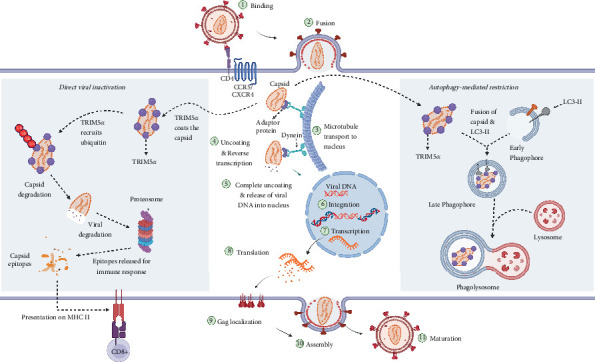
Mechanisms for HIV restriction mediated by TRIM5*α*. The direct viral inactivation mechanism involved binding of TRIM5*α* to the incoming viral capsid, causing premature uncoating and subsequent proteosomal degradation. In the absence of TRIM5*α*, HIV goes through all stages of the replication cycle, producing infectious virions, which are released and infect new cells. The autophagy-mediated restriction mechanism involves binding of the TRIM5*α* viral capsid, facilitating fusion with LC3-II and subsequent degradation by lysosomal enzymes. Created in BioRender (https://app.biorender.com/).

TRIM5*α* inhibits HIV-1 in old world monkey (OWM) cells by targeting the viral CA [70]. This molecule mediates viral restriction at early postentry stage in a species-specific manner by interacting with viral CA through its PRYSPRY/B30.2 domain [70] (Figure 4). Human TRIM5*α* have limited anti-HIV-1 activity in vivo, whereas rhesus macaque TRIM5*α* and TRIM5-CypA fusion are highly effective against primate lentiviruses [71]. The human TRIM5*α* potently restrict the N-tropic murine leukemia virus (N-MLV) and appears to moderate HIV-2 [72] infection, potentially contributing to the attenuated disease course in HIV-2 infected individuals [59, 73, 74].

In absence of TRIM5*α*, HIV CA, uncoating occurs following either of the two models suggested [75–78]. In the first model, upon the viral core entry, its CA shell remains intact until it reaches and docks on the nuclear pore. The CA then disassembles as viral DNA is imported into the nucleus [76]. In the second model, the CA undergoes structural remodeling while in the cytoplasm resulting in rapture or loss of integrity of the CA shell. However, a portion of the CA remains intact until complete disassembly at the nuclear pore [75].

A well-coordinated balance between uncoating and reverse transcription is necessary for a successful infection to occur. Studies have revealed that mutation in CA that either increase or reduce the intrinsic CA stability causes significant loss of infectivity, which is associated with defect in production of viral reverse transcripts [79]. Also, inhibition of reverse transcription has been shown to delays the onset of uncoating [80, 81]. Since TRIM5*α* induces aberrant uncoating and inhibits reverse transcription, understanding the relationship between these post entry processes would provide insights into the mechanism of TRIM5*α*-mediated viral restriction. Wu and colleagues demonstrated the inhibitory activity of TRIM5*α* against HIV-2, which was characterized by increased CD4^+^ T-cells and longer AIDS-free survival [82].

Another mechanism potentially involves TRIM5*α*-induced autophagy-mediated restriction through binding to CA and causing it to fuse with LC3-II phagophore (Figure 4). Subsequently, the phagophore fuses with lysosome forming a phagolysosome that triggers lysosomal enzymes to break down the CA [83]. TRIM5*α* slows down disease progression among HIV-2 infected individuals [72]. Moreover, some genetic variants of TRIM5*α* have been associated with an enhanced HIV-1 restriction activity [84]. In this regard, it is possible to leverage gene therapy to enhance antiviral activities of TRIM5*α* [85].

### 4.5. Virus–Virus Interaction

Interaction between HIV-1 and HIV-2 at the genetic level can explain the slowed disease progression phenotype during dual infection. Factors such as transactivation response (TAR) elements from HIV-1 and HIV-2 are known to compete for Tat binding, influencing transcription initiation [24]. Also, during reverse transcription, HIV-1 and HIV-2 can exchange their genetic materials, resulting in mosaic viruses [86]. The potential role of these interactions in slowing disease progression has been discussed in the ensuing section.

#### 4.5.1. HIV-2 Transactivation Response (TAR-2) Element Compete With HIV-1 TAR-1 for Tat Binding

**Figure 5:**
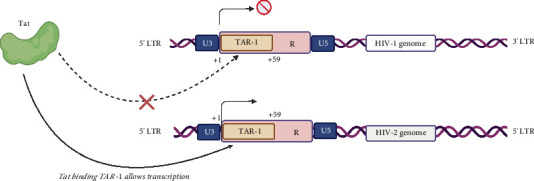
Competition of tat binding between HIV-1 and HIV-2 TAR elements (TAR 1 and TAR 2, respectively). TAR 2 has a higher affinity for tat, hence depriving TAR-1 of Tat stalling the transcription initiation process in HIV-1. Created in BioRender (https://app.biorender.com/).

HIV-2 genetic elements can potentially dampen HIV-1 replication in HIV-dual infections. Recently, it was shown that TAR-2 element can dampen HIV-1 transcription [24]. TAR-1 RNA decoys have previously been used to competitively inhibit HIV-1 Tat-mediated transcriptional activation [87]. Tat-1 can transactivates HIV-2 gene expression via TAR-2 element, despite slight functional differences with Tat-2. Thus, when TAR-2 is present during dual infection, it competes with TAR-1 for Tat-1 binding resulting in inhibition of HIV-1 transcription [24]. HIV-1 replication is thus reduced as a result of competitive binding of Tat-1 to TAR-2, reducing its availability to bind to TAR-1 [24] as shown in Figure 5. Furthermore, in vitro and in vivo functional studies suggest an interaction between at least two of the hairpin structures of TAR-2 with Tat-1, demonstrating a higher affinity of interaction that would increase the effectiveness of a TAR-2 RNA binding relative to TAR-1 [88]. Therefore, exploring the use of TAR-2 RNA decoys to block Tat-1-mediated HIV-1 transcription would help to reduce HIV-1 disease progression.

#### 4.5.2. Genetic Recombination

Recombination is a common feature among retroviruses that ensures generation of diversity during viral replication [89]. Initial steps of HIV replication involve conversion of a single-stranded genomic RNA into a double-stranded complementary DNA. During this process, the polymerase enzyme utilizes both RNA templates, generating a mosaic cDNA carrying fragments from both RNA strands [86]. Both HIV-1 and HIV-2 intersubtype recombinants have been described [90, 91]. Currently, more than 118 circulating recombinant forms have been listed on the Los Alamos HIV database (https://www.hiv.lanl.gov/components/sequence/HIV/crfdb/crfs.comp). Some HIV-1 recombinants form dominant epidemic lineages, including CRF01_AE circulating in Thailand and China, while CRF02_AG dominates the West and Central Africa epidemics [91, 92].

Some subtypes of HIV-1, such as Subtype C, are more infectious with higher transmission rates compared to others such as CRF02_AG, which remain geographically constrained to West Africa. Host genetics, transmission bottlenecks, social/behavioral and environmental factors could contribute to the variable transmission of different subtypes [93]. Estimated in vivo HIV recombination rates range between 1.4 × 10^−5^ to 2 × 10^−4^ breakpoints per site per generation [94], which is lower than that reported *in vitro* [95]. Studies on HIV recombination primarily focus on recombinants arising from HIV-1 intersubtype recombination events. However, in West and Central Africa where both HIV-1 and HIV-2. However, some regions, such as West and Central Africa, harbor both HIV-1 and HIV-2, which cocirculate, and dual infections occur. Dually infected individuals could harbor recombinants of HIV-1 and HIV-2 [40]. Interestingly, to date, it is not known whether these viruses recombine in vivo. There is no unequivocal evidence of recombination in HIV-dual infection. Only in vitro recombination between HIV-1 and HIV-2 has been demonstrated during dual infection [96], which may not be directly translated to imply formation of these recombinants during natural HIV-dual infections.

Phylogenetic evidence of recombination among retroviruses showed high frequency of recombination events [97]. Historically, recombination between distinct SIV isolates resulted in formation of novel SIVs that infect chimpanzees (SIV_cpz_) [97]. These recombinants were the precursor of HIV-1 [98]. SIV_cpz_ is a chimera, carrying pol gene from SIV that infects red-capped mangabeys and env gene from SIV that infects greater spot-nosed monkeys. It is likely that SIV_cpz_ emerged from recombination that occurred in chimpanzees infected with SIV that infect red-capped mangabeys and SIV that infects greater spot-nosed monkeys generating a novel chimera [96]. Subsequent recombination events in chimpanzees ultimately resulted in formation of HIV-1.

Recombination between HIV-1 and HIV-2 has been demonstrated *in vitro* by Rawson and colleagues [99]. In another study, Motomura and colleagues used GFP-tagged near full-length HIV genomes with GFP inactivating mutations that were repaired upon recombination between HIV-1 and HIV-2 [96]. Overall, they identified recombinant genomes with heterologous long terminal repeats (LTRs) [96]. While there is evidence of potential recombination events between HIV-1/2 in vitro, the impact of such recombination on disease progression and clinical outcomes has not been investigated. We hypothesize that the resultant HIV-1/HIV-2 recombinants could be less aggressive compared to HIV-1, contributing to the slow disease progression phenotype observed among dually infected individuals.

## 5. HIV-Dual Infections and Implications for Therapy

The HIV epidemic in West and Central Africa is less aggressive yet these regions harbor both HIV-1 and HIV-2. In other regions where HIV-2 has not been reported such as South and Eastern Africa, the epidemics are more aggressive. Thus, we can hypothesize that HIV-2 contributes to the reduction of infectivity or replication rate of HIV-1. However, testing this hypothesis is compounded by the limited access to active HIV-2 cases, while *in vitro* studies are hindered by the poor infectivity of cell lines by HIV-2. Nonetheless, exploring the intricacies of HIV-1 and HIV-2 interactions would provide potential leads to new drug and/or vaccine targets. For instance, understanding the mechanism through which HIV-2 Vpx inhibits HIV-1 cell invasion as demonstrated by Mahdi and colleagues [43] can open new avenues for design of inhibitory small molecules against HIV-1.

Additionally, by leveraging the CA degradation mechanism of TRIM5*α*, we can gain insights into the development of blockers that target the incoming CAs and destroy them before an infection is established. This approach could serve as a good preventive approach, inhibiting the virus at the early postentry stages. Such a drug would be a good supplement for the currently available entry inhibitors. Following exemplary performance of the CA inhibitor—lenacapavir during PURPOSE II clinical trial [100], it is envisioned that the CA holds the potential for a cure. Moreover, the CA is unique to the virus, and their inhibitors would be highly selective against the virus. By targeting the more conserved portions of the CA such as PRYSPRY/B30.2 binding domains [70], these inhibitors would be type, subtypes and strain transcending. Other CA targeting compounds in clinical trial targeting the capsid-C-terminal domain (CA-CTD) and capsid-N-terminal domain (CA-NTD) could be critical in disrupting CA assembly and function [101]. These CA inhibitors have shown varying degree of HIV-1 inhibition but have not been tested against HIV-2 [102].

The concept of inhibiting HIV-1 viral replication by TAR^HIV-2^ has been studied [87]. To date, TAR^HIV-1^ RNA decoys have been developed and used to inhibit HIV-1 transcription, but recent data suggest that TAR^HIV-2^ are likely more effective at inhibiting HIV-1 replication [24]. These RNA-based HIV inhibition strategies would unveil newer drugs with high specificity against the viral TAR, stalling viral transcription complex. For instance, several HIV transcription inhibitors that bind to viral RNA have been suggested [103].

## 6. HIV-2-Specific Treatment Challenges

Due to considerable genetic differences between HIV-1 and HIV-2, differential sensitivity of HIV-2 to ARTs developed for HIV-1 has been recorded [104]. Due to the lack of HIV-2-specific ART, treatment of HIV-2 and HIV-dual infections relies on drugs developed for HIV-1. Whereas HIV-1 and HIV-2 share genomic architecture and sequence homology, there are substantial differences in some of the proteins targeted by ARVs. These differences could confer HIV-2 with intrinsic resistance to some of the ARV drugs [105]. Indeed, the sensitivity of HIV-2 to nonnucleoside reverse transcriptase inhibitors (NNRTIs) and protease inhibitors is poor [106], and there is conflicting data about the effectiveness of integrase strand transfer inhibitors (INSTI) in HIV-2 [107]. Whereas INSTI have shown remarkable control of HIV-1, clinical trials have reported differential sensitivity of HIV-2 to INSTI compared to HIV-1 [108]. Some studies have reported potential intrinsic resistance of HIV-2 against some INSTI [109]. The scarcity of HIV-2 sequence data suggests limited genetic surveillance of HIV-2 drug resistance, perhaps due to limited access to sequencing tools in resource-limited settings [110].

## 7. Immune Responses in HIV-1 and HIV-2 Coinfection and Insights for a Cure

HIV-1 and HIV-2 are strikingly similar in genetic and biological properties, including genome structure, cell tropism, and immune cell depletion, yet HIV-2 exhibits a longer clinical latency period and a significantly lower rate of disease progression and transmission [111]. The distinct differences in the pathogenicity of HIV-1 and HIV-2 provide a basis for exploring immunotherapy. Thus, by understanding the immune landscape in HIV-1 and HIV-2 mono and dual infections, we can identify immune responses that are possibly involved in restricting the rate of disease progression during HIV-2 infection and design an immunotherapy to potentiate similar immune responses.

## 8. Bottlenecks to HIV-2 and HIV-Dual Studies

Due to the lower relative contribution of HIV-2 to the global HIV pandemic, it receives limited research funding. Surprisingly there is no generally accepted in vitro infection model for HIV-2 unlike HIV-1. Additionally, due to the low transmission capacity of HIV-2, lack of continuous monitoring, and low prevalence, most HIV-dual infection use small cohort sizes, which hinders the application of findings from such studies. Moreover, there is a paucity of clinical evidence for HIV-1 and HIV-2 recombination among HIV-dual infections. Whether these recombinants exist and their impact on HIV epidemic remain largely unknown.

## 9. A Call to Action

Although HIV-2 causes a mild infection, its impact on HIV-1 is not fully understood. Future studies should focus on developing efficient animal models that will promote mechanistic studies of interactions between HIV-1 and HIV-2 during dual infections. Leveraging the knowledge gained from mechanisms of interactions between HIV-1 and HIV-2 would provide alternative avenues for developing curative therapies. For instance, exploring the potential for developing immunotherapy that could induce *β*-chemokine production in a similar fashion as HIV-2 would achieve viral suppression and better immune outcomes without the conventional ART. Another promising avenue is the role of HIV-2 TAR in RNA-based gene therapy. The capacity of HIV-2 Vpx to inhibit HIV-1 is yet to be determined; yet, it holds potential for curative therapy. Moreover, there is a need to enhance genomic surveillance of viral isolates from dually infected individuals to track the evolution of HIV-1 and HIV-2 in the context of dual infections.

## 10. Conclusion

To the best of our knowledge, this is the first systematic integration of HIV-1/HIV-2 interaction mechanisms and their implications for future therapeutic strategies. Novel treatment approaches could leverage HIV-1/HIV-2 interactions such as TAR RNA–based gene therapy or Vpx-targeted treatments. A deeper understanding of these mechanisms has the potential to revolutionize HIV cure.

## Figures and Tables

**Table 1:** The difference and similarities between HIV-1, HIV-2, and HIV-dual infections.

	**Variables**	**HIV-1 infection**	**HIV-2 infection**	**HIV-dual infection**	**Reference**
1	CD4+ T-cells decline rate	Faster decline rate (0.9% per year) over time	Half the rate HIV-1 (0.4% per year) over time	Decline is moderately slower than HIV-1 but faster than HIV-2	[13]
2	Viral loads	High viral loads	Lower viral loads, about 28-fold lower than in HIV-1 infection	HIV-1 viral loads are lower compared to HIV-1 monoinfection	[13, 33]
3	Length of asymptomatic phase	Shorter asymptomatic phase about 8.2 years	Longer Asymptomatic phase about 15.6 years	Asymptomatic phase is prolonged compared to HIV-1	[13, 33, 34]
4	Vertical transmission	High perinatal transmission of HIV-1 (15-30%) among HIV-1 infected mothers	Lower perinatal transmission of HIV-2 in infected mothers (1-2%)	—	[35]
5	Pretreatment viral loads	High pretreatment viral loads	Lower pretreatment viral loads	—	[36].
6	Viral diversity	High	Low	—	[37–39]
7	Geographical distribution	HIV-1 has reached epidemic proportions throughout the world	Largely restricted to West and Central Africa with few cases reported in Americas, Europe, and Asia	Similar to HIV-2	[40–42]
8	Transmission route	Predominantly through sexual contact and perinatally and other less common routes including blood transfusion, sharing of sharp objects and body fluids	Share same transmission route with HIV-1	Transmission can be either simultaneous or sequential through same route as monoinfections	[32]
9	Target-cells	Primarily infect the CD4+ T-cells	Infect CD4+ T-cells. In addition can infect myeloid cells including monocytes and macrophages with higher efficiency than HIV-1 due to presence of Vpx, which degrades SAMHD1.	Same target cells	[43]
10	Pathogenesis	Depletes CD4^+^ T-cells at a relatively higher rate	CD4^+^ T-cell depletion is slower	Depletes CD4^+^ T-cells slightly slower than in HIV-1 monoinfection	[44]
11	Immune activation	Higher tendency of activating CD4^+^ T-cells rendering them highly susceptible to infection	Can slow down immune activation through HIV-2 Nef protein, which downregulates TCR-CD3 complex formation	Immune activation is lower compared to HIV-1 monoinfection	[45]

## Data Availability

Data sharing is not applicable to this article as no new data were created or analyzed in this study.
